# Comparison of Radiography and Ultrasonography for Diagnosis of Diaphragmatic Hernia in Bovines

**DOI:** 10.4061/2010/939870

**Published:** 2010-02-21

**Authors:** Hakim Athar, Jitender Mohindroo, Kiranjeet Singh, Ashwani Kumar, Mulinti Raghunath

**Affiliations:** Department of Surgery and Radiology, College of Veterinary Science, Guru Angad Dev Veterinary & Animal Sciences University, Ludhiana, Punjab 141 004, India

## Abstract

The present study was conducted on 101 animals suffering from thoracoabdominal disorders; out of which twenty seven animals (twenty six buffaloes and one cow) were diagnosed with diaphragmatic hernia based on clinical signs, radiography, ultrasonography, and left flank laparorumenotomy. Radiography alone confirmed diaphragmatic hernia in 18 cases (66.67%) with a sac-like structure cranial to the diaphragm. In 15 animals the sac contained metallic densities while in three cases a sac-like structure with no metallic densities was present. Ultrasonography was helpful in confirming diaphragmatic hernia in 23 cases (85.18%) and ultrasonographically reticular motility was evident at the level of 4th/5th intercostal space in all the animals. B+M mode ultrasonography was used for the first time for diagnosis of diaphragmatic hernia in bovines and the results suggested that ultrasonography was a reliable diagnostic modality for diaphragmatic hernia in bovines.

## 1. Introduction

Diaphragmatic hernia is defined as the passage of abdominal viscera into the thoracic cavity through a congenital or acquired opening in the diaphragm at the musculotendinous junction. It is a chronic wasting and inflammatory thoracoabdominal disorder in adult milk buffaloes [1, 2] and has also been reported in cows [3–5] and buffalo bulls [6]. Diaphragmatic hernia is observed mostly in adult dairy animals that are either in their late gestation or have recently calved [7, 8]. Diaphragmatic hernia may occur as a result of trauma, parturition, or progressive weakening of the diaphragm adjacent to a hardware perforation and reticuloperitonitis [9]. The main cause of diaphragmatic hernia in buffaloes has been reported to be foreign body syndrome while other conditions that increased intra-abdominal pressure, for example, ruminal tympany, violent fall, advanced pregnancy, parturition process, chronic cough, and straining due to any reason could act as an exciting factor [10].

There have been reports on the use of ultrasonography for the diagnosis of diaphragmatic hernia in bovines [5, 11]. However no reports are available on the comparison of ultrasonography and radiography for the diagnosis of diaphragmatic hernia in bovines. The present study was conducted to compare the efficacy of ultrasonography and radiography to diagnose diaphragmatic hernia in bovines. 

## 2. Materials and Methods

### 2.1. Animals

The present study was conducted on 101 bovines presented with thoracoabdominal disorders to the Department of Veterinary Surgery and Radiology, GADVASU during the period between April 2008 and March 2009. Their history, age, sex, breed, duration of illness, feed intake, rumination, defecation, pregnancy status, milk yield, signs of pain, coughing, regurgitation, and presence/absence of tympany were recorded. Rectal temperature, heart rate, respiration rate, rumen motility, rumen fluid pH were also noted and rectal examination was also performed in all the cases.

### 2.2. Radiography

Reticular radiographs in right lateral recumbency with forward stretched forelimbs at the end of inspiration were taken. Survey radiographic evaluation consisted of one 14 inch × 17 inch radiograph of the reticulum using Siemens X-ray machine having maximum mAs of 400 and KVP of 150. The exposure factors used for the present study were 50–60 mAs, 90–100 KVP at a film focal distance of 90–110 cm. 

### 2.3. Ultrasonography

Ultrasonography was performed on the standing animals restrained in a cattle crate without any sedation. The Concept/MCV Veterinary Ultrasound Scanner (Dynamic Imaging), in real-time B-mode with a 3.5 MHz microconvex transducer, was used which was easy to manoeuvre in the narrow intercostal spaces. Right lateral wall of the thorax from 3rd to 8th intercostal space was shaved, cleaned and ultrasound gel was applied liberally for optimum transmission of ultrasound waves. The motility of reticulum was first noted in the abdominal cavity by placing the transducer at the level of right elbow in the sixth or seventh intercostal spaces and then moving it ventrally [11]. The reticulum was located, observed for three minutes without moving the transducer and the frequency of biphasic reticular contractions was recorded to give the reticular motility pattern [12]. Thereafter, the reticulum was located in the thoracic cavity by placing the transducer in the intercostal spaces of fifth, fourth, or third rib at level of elbow and then moving ventrally and measurements of reticular contractions were assessed. Both B and B+M modes were used to evaluate the reticulum. The reticular motility in the abdominal cavity was compared with that of the thoracic cavity.

### 2.4. Laparorumenotomy

Left flank Laparorumenotomy was performed under linear infiltration anaesthesia in standing animals. The ultrasonographic and radiographic findings were confirmed upon laparorumenotomy.

## 3. Results

Diaphragmatic hernia was diagnosed in twenty seven cases out of 101 cases of thoracoabdominal disorders presented to the clinics based on radiography, ultrasonography, and laparorumenotomy. The animals suffering from diaphragmatic hernia had mean age of 6.30 ± 0.37 years with age ranging from 2.5 to 10 years. The duration of illness ranged from six days to four weeks with a mean of 21.04 ± 5.21 days. Fifteen (55.55%) animals were dull and depressed at the time of presentation and had a dry muzzle while the others appeared alert. Fourteen (51.8%) animals had recently parturated, six (22.2%) were in advanced stage of pregnancy, while rest of the animals were in different stages of lactation. Twenty animals had been treated unsuccessfully with antibiotics and antibloat agents while in the rest of the animals no treatment was given. Nineteen (70.4%) animals were passing hard, black faeces; seven (25.9%) animals had reduced faecal output while one animal was passing loose faeces. Recurrent tympany was observed in majority of the animals (63%) while three animals had persistent tympany and five did not show any tympany. Two animals had a single episode of tympany. Regurgitation was observed in two cases. Rumen was hyper motile (4.88 ± 0.21/2 minutes) but with reduced strength of ruminal contractions in seventeen (62.9%) cases while in three animals rumen was hypomotile (2.00 ± 0.00/2 minutes). No rumen motility could be appreciated in four animals while in three animals rumen motility was (3.00 ± 0.00/2 minutes). Brisket oedema was observed in two animals. Rectal examination revealed mushy rumen in all but seven cases. Rumination was absent in seventeen animals while ten animals were ruminating irregularly. The mean rectal temperature of animals was 38.76 ± 0.15°C. Respiratory rate and heart rate were within normal range in all the animals. 

**Figure 1:**
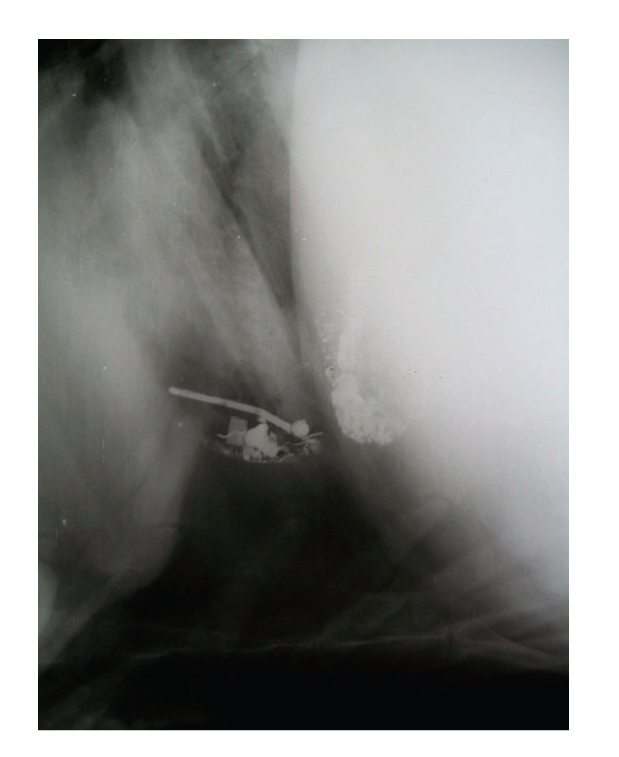
Lateral radiograph of reticulum showing clearly demarcable diaphragmatic line and presence of potential and nonpotential metallic densities cranial to diaphragm.

**Figure 2:**
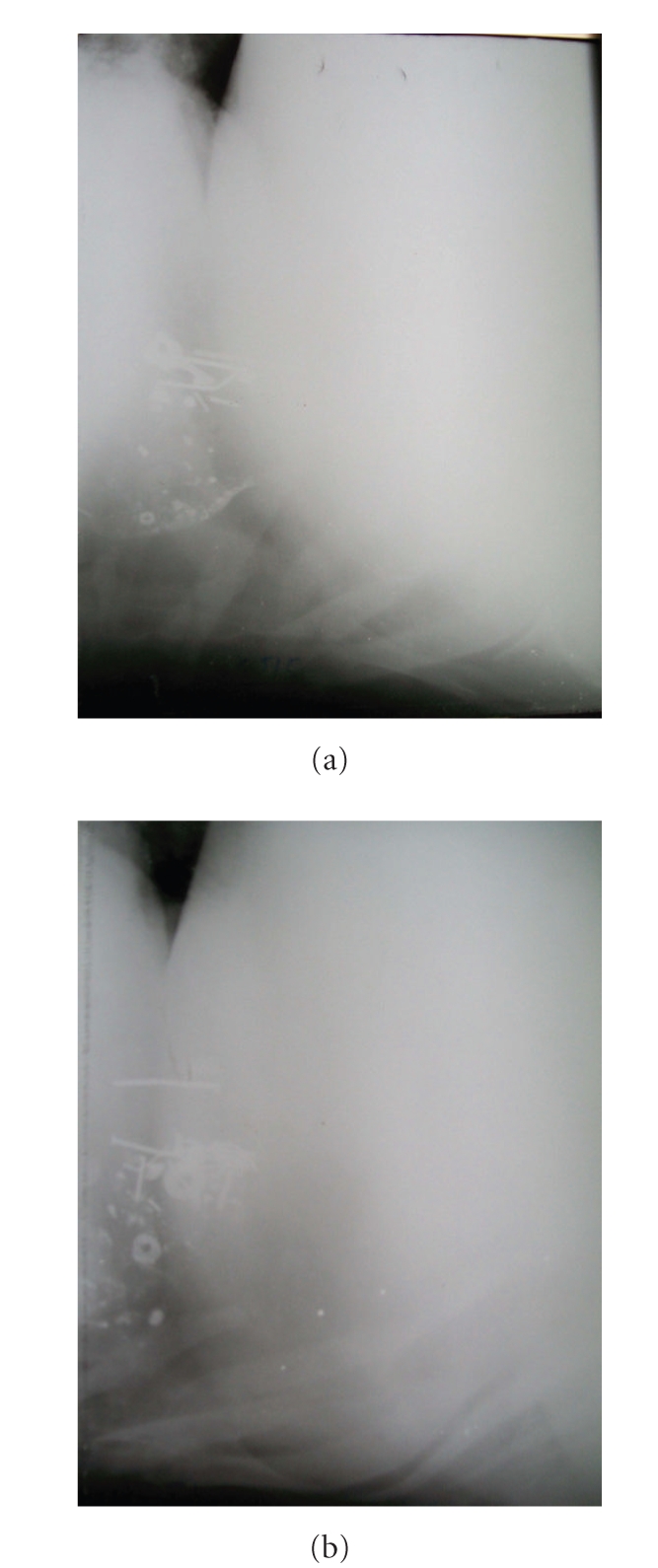
Lateral radiographs of reticulum showing indistinct diaphragmatic line and presence of metallic densities in a sac-like structure cranial to diaphragm.

**Figure 3:**
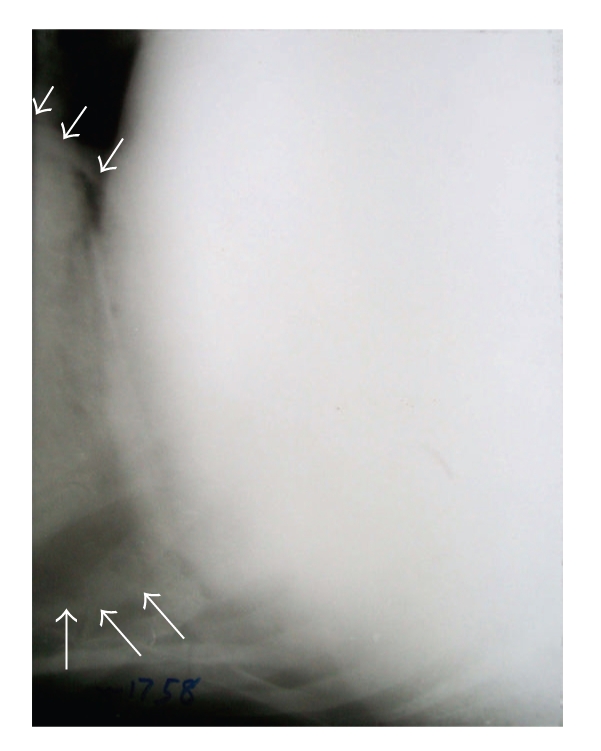
Lateral radiograph of reticulum showing unclear diaphragmatic line and a sac-like structure (arrows) of soft tissue density cranial to diaphragm.

**Figure 4:**
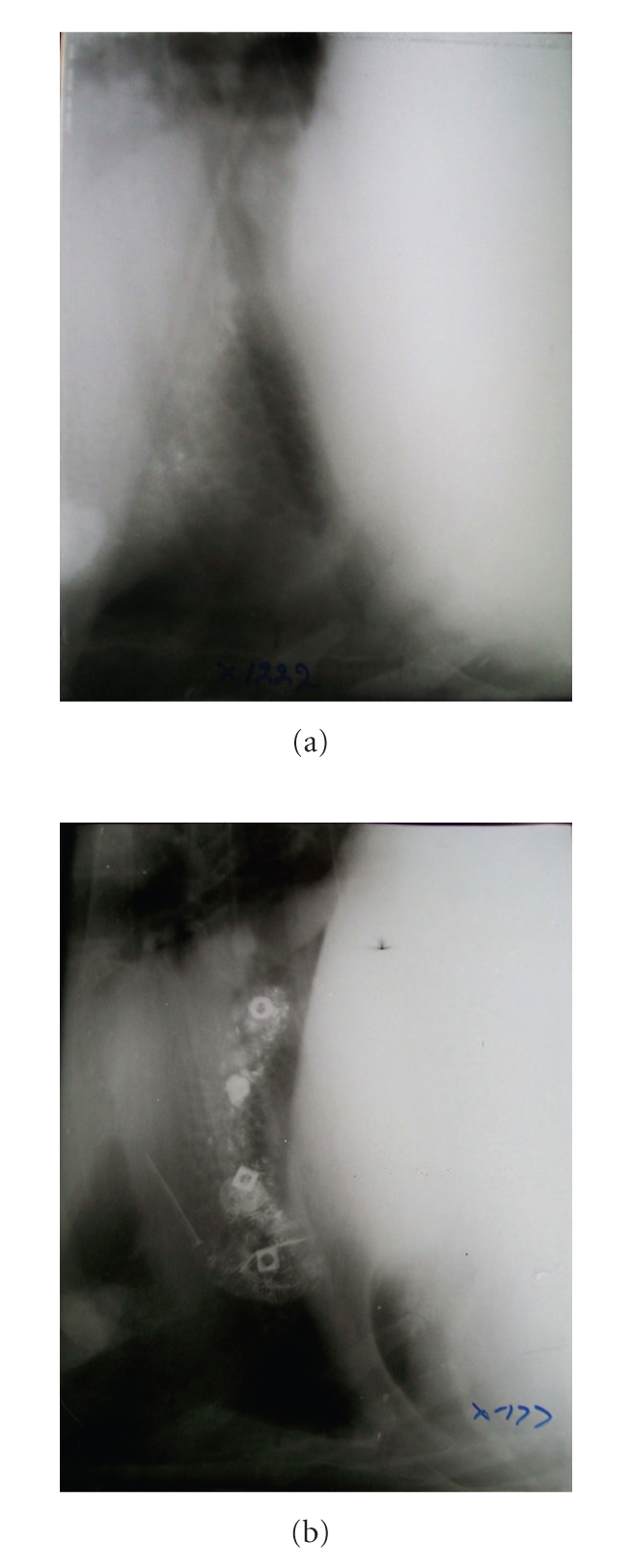
Lateral radiographs of reticulum showing honey comb reticular pattern of the sac-like structure in the thoracic cavity without (a) or with (b) metallic densities.

**Figure 5:**
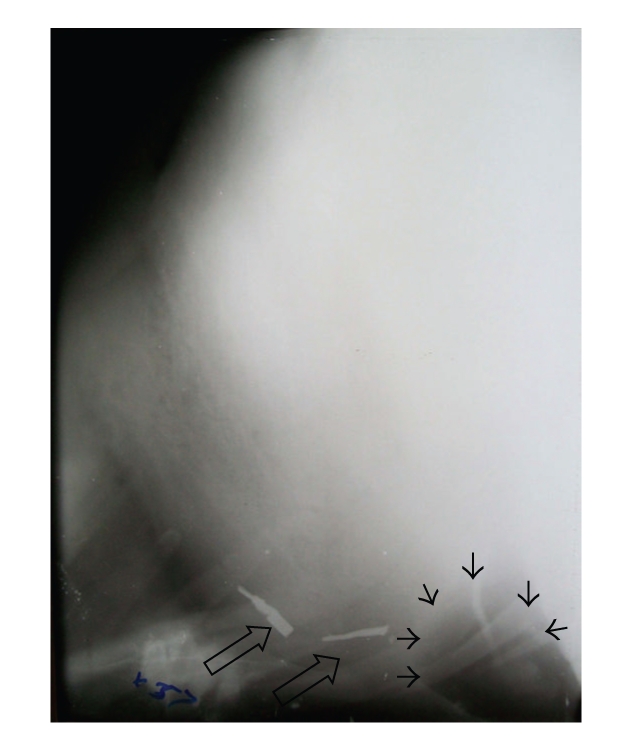
Lateral radiograph of reticulum showing indistinct diaphragmatic line. Metallic densities (hollow arrows) seen inside thoracic cavity. Note the gas density (black arrows) having embedded metallic density suggesting reticular abscess.

**Figure 6:**
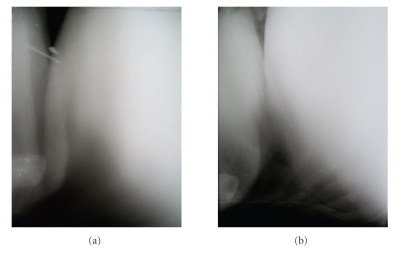
Lateral radiographs of reticulum showing indistinct line of diaphragm. Diaphragmatic hernia is not apparent on radiographs.

Radiographic examination of the reticulum performed in right lateral recumbency revealed a break in the continuity of diaphragmatic line in twenty cases. In fifteen cases a sac-like structure cranial to the diaphragm with metallic densities was evident (Figures 1, 2(a), and 2(b)). In three cases only sac-like structure without any metallic densities cranial to the diaphragm was present (Figure 3). All these eighteen animals were declared positive for diaphragmatic hernia on the basis of radiography. Among these eighteen cases, reticular honey comb pattern was visible in the thoracic cavity in two animals (Figures 4(a) and 4(b)) and in one animal a gas density ventral to the reticulum was evident suggestive of reticular abscess (Figure 5). In the rest of the animals, diaphragmatic hernia could not be diagnosed on the basis of radiography although a break in continuity of diaphragm was observed on the radiographs (Figures 6(a) and 6(b)). 

**Figure 7:**
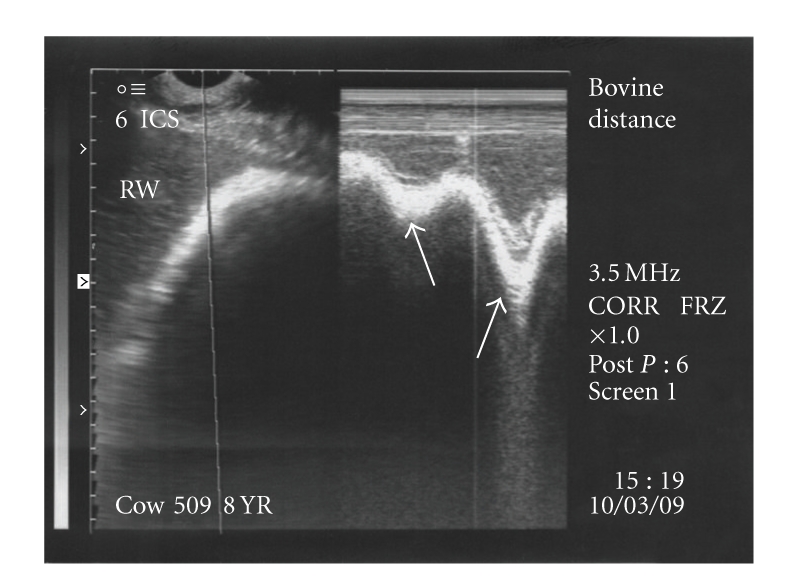
Partial biphasic reticular motility (arrows) seen at the level of 6th intercostal space on B+M mode ultrasonogram.

Reticulum was scanned as a smooth crescent shaped structure with biphasic but reduced reticular motility in seventeen animals (Figure 7); six animals had complete biphasic reticular motility while in four animals reticular motility was not present. 

**Figure 8:**
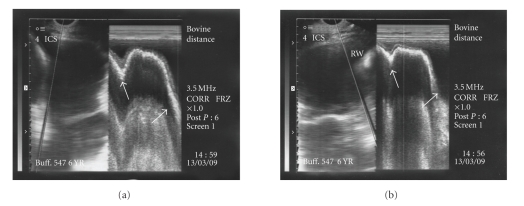
Complete biphasic reticular motility (arrows) evident in the thoracic cavity at the level of 4th intercostal space seen on B+M mode ultrasonograms confirmatory for diaphragmatic hernia.

**Figure 9:**
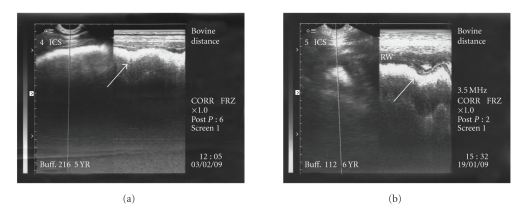
Gliding reticular motility in the thoracic cavity cranial to the 5th intercostal space visualized on B+M mode ultrasonograms (arrow) confirmatory for diaphragmatic hernia.

**Figure 10:**
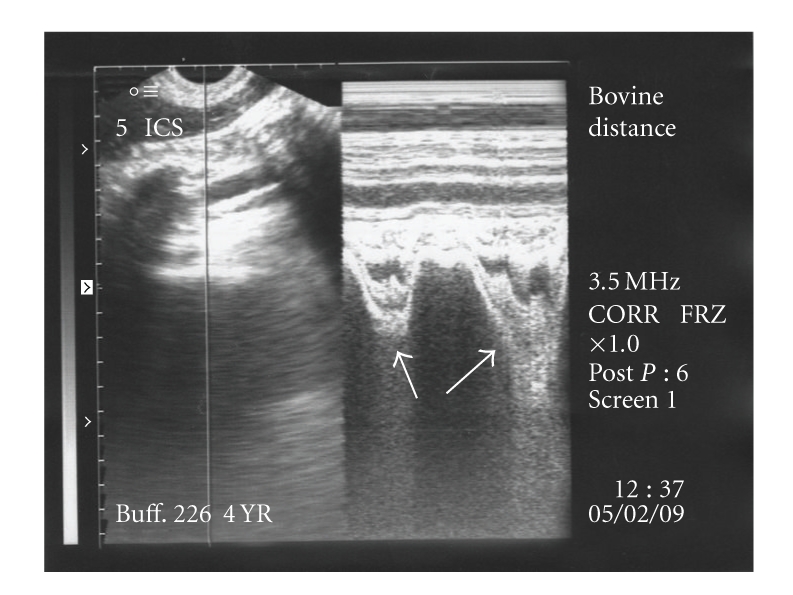
Reduced biphasic reticular motility (arrows) seen in the thoracic cavity on B+M mode ultrasonograms.

The reticulum was further scanned in the thoracic cavity at 4th-5th intercostal spaces where it appeared as a relatively straight line. In six animals, complete reticular motility was present in the thoracic cavity (Figures 8(a) and 8(b)) characterised by biphasic pattern. Gliding and incomplete reticular motility were recorded in three cases (Figures 9(a) and 9(b)) and in fourteen animals, reduced biphasic reticular motility was evident (Figure 10) within the thoracic cavity. All these twenty three animals were declared positive for diaphragmatic hernia based on ultrasonographic findings. The scanning of reticulum with motility cranial to 5th intercostal space was considered confirmatory for declaring the case positive for diaphragmatic hernia. In the remaining four animals, no reticular motility could be identified in abdominal or thoracic cavity although a reticular wall-like structure without any motility was visible in the thoracic cavity at the level of 4th intercostal space suggesting possibility of diaphragmatic hernia. Ultrasonographically, the honey comb pattern of the reticulum and foreign bodies inside the reticulum were not evident in any animal. In two animals reticular motility was observed at the level of 5th intercostal space. These were declared positive for diaphragmatic hernia but the hernia was ruled out on subsequent laparorumenotomy. These two false positive cases were not included in the twenty seven cases of diaphragmatic hernia in the present study. However, no false negative diagnosis of diaphragmatic hernia was made on ultrasonography. Reticular abscess was visualized along with diaphragmatic hernia in three animals. The abscess was seen at the level of sixth and seventh intercostal spaces adjacent to the reticular wall characterized by echogenic capsule of varying thickness with hypoechogenic contents. 

 Left flank laparorumenotomy was performed in twenty three animals and reticulo diaphragmatic hernia was confirmed in all cases with hernial ring varying in size from 10 cm to 20 cm. In one buffalo two hernial rings were present. Hernial rings were present on the right hemidiaphragm in all the cases. In twenty animals, contents of rumen and reticulum were mushy and were overfilled with frothy contents in fifteen cases. In three cases contents of the rumen were normal. Reticular abscess was present in three cases. Herniated part of the reticulum was adhered all around the ring in twenty animals while in three animals the herniated reticulum was free of adhesions and could be pulled back through the ring. Four cases diagnosed positive both with ultrasonography and radiography were not subjected to laparorumenotomy due to the unwillingness of the owner and the diagnosis was confirmed based on ultrasonography and radiography only.

## 4. Discussion

Diaphragmatic hernia has been considered as a serious digestive disorder of buffaloes [2, 10]. In the present study 26 buffaloes and one cow had diaphragmatic hernia. Higher prevalence of diaphragmatic hernia in buffaloes versus relatively lower prevalence in cows may be attributed to the lesser collagen content, elasticity, and vascularity of buffalo diaphragm [10]. They also observed that habit of wallowing found among buffaloes may also be an exciting factor for the rupture of diaphragm. The clinical signs of partial anorexia with suspended rumination, recurrent tympany, scant faeces were in accordance with those reported previously [10, 13]. Recurrent tympany in cases of diaphragmatic hernia has been also reported [5]. Interference of rumen peristalsis owing to reticular adhesions was regarded as major cause of tympany [14]. 

Diagnosis of diaphragmatic hernia is based upon the clinical examination, plain and contrast radiography, laparorumenotomy [15–17]. Historically, radiographs have been considered as the best means of confirming the diagnosis of diaphragmatic hernia and may help to differentiate it from other allied conditions [18]. The normal outline of heart and diaphragm may be obscured and the foreign objects in the reticulum may be seen in the thorax as this is the most commonly involved organ and the honey comb or foreign objects in the reticulum may be seen in the thorax [19, 20]. Radiographically the presence of honey comb pattern of the herniated reticulum inside the thoracic cavity has been reported earlier [17] which may be due to accumulation of gas resulting from fermentation in the reticular area [21]. A good success rate (66.67%) in radiographic diagnosis of diaphragmatic hernia was similar to earlier reports [22]. In contrast survey radiography in left lateral recumbency has been considered sufficient for diagnosis of diaphragmatic hernia and shape size and radiopacity of hernial swelling can easily be determined [17]. Pleuritis and other masses such as tumours and abscesses can also mimic hernia on plain films [19]. 

A 3.5 MHz microconvex transducer was found adequate for scanning the reticulum both in the abdominal as well as thoracic cavities [11]. However a 3.5 MHz linear transducer has been advocated for scanning of reticulum in cattle [12, 23–25]. The normal reticulum appears as a half-moon shaped structure and contracts at regular interval once per minute in a biphasic manner first of which is incomplete [12]. The biphasic but reduced reticular motility in the abdominal cavity could be attributed to herniation of a part of reticulum into the thoracic cavity and reticulophrenic adhesions [26]. In the present study complete/gliding/reduced reticular motility inside the thoracic cavity confirmed the diaphragmatic hernia. The incomplete gliding motility and the reduced motility of reticulum in the thoracic cavity may be attributed to the perireticular adhesions that form subsequent to diaphragmatic hernia [2]. A high percentage of success in diagnosis of diaphragmatic hernia by ultrasonography suggested that ultrasonography was more reliable than radiography in making a correct diagnosis of the disease. Reticular motility within the thoracic cavity was considered important criteria to diagnose diaphragmatic hernia. Therefore four animals could not be declared positive for diaphragmatic hernia despite amotile reticular wall being scanned at the 4th intercostal space. Inability to visualise honey comb pattern and metallic foreign bodies in the reticulum may be attributed to presence of gas in the reticulum [11, 24]. The two animals which were diagnosed falsely for diaphragmatic hernia were having extremely distended abdomen and were subsequently diagnosed for vagal indigestion. In these cases, possibly due to extreme pressure on diaphragm the reticulum could have been scanned at the 5th intercostal space. Ultrasonographic features of reticular abscess recorded in the present study were similar to those observed by earlier workers [24]. Laparorumenotomy confirmed diaphragmatic hernia in all the animals operated and has been advocated as the best method to confirm diaphragmatic hernia in bovines. The presence of hernial ring on the right hemi diaphragm is consistent with the earlier findings [22]. The extreme right side location of the hernial ring facilitated easy access via ultrasonography and made it possible to diagnose diaphragmatic hernia from the right side. The mushy and frothy contents of rumen in cases of diaphragmatic hernia may be due to the entrapment of reticulum inside the thoracic cavity that hinders the normal reticular movements and distorts the oesophageal groove and reticuloomasal orifice producing motility disturbances reflected by hypermotility of the rumen, generation of frothy rumen contents, persistent or moderate tympany, and overfilling of the rumen [8]. The perireticular adhesions and reticular abscess diagnosed by ultrasonography were confirmed on laparorumenotomy. Ultrasonography was less stressful for the animals than radiography because it could be performed on the standing animals without any discomfort to the patient and did not involve use of harmful radiations. For radiography it was essential to restrain the animals in lateral recumbency which was stressful for the patients, especially the ones in late pregnancy.

Based on the findings of this study, it was concluded that ultrasonography was more accurate than radiography in the diagnosis of diaphragmatic hernia in cows and buffaloes with less discomfort for the patient. However, making false positive diagnosis cannot be ruled out.
